# Transgenic line for the identification of cholinergic release sites in *Drosophila melanogaster*

**DOI:** 10.1242/jeb.149369

**Published:** 2017-04-15

**Authors:** Katarina Pankova, Alexander Borst

**Affiliations:** 1 Max Planck Institute of Neurobiology, 82152 Martinsried, Germany; 2 Graduate School of Systemic Neurosciences, LMU Munich, 80539 Munich, Germany

**Keywords:** VAChT, Acetylcholine, Neurotransmitter, Motion vision, Mi1 neurons, Tm3 neurons

## Abstract

The identification of neurotransmitter type used by a neuron is important for the functional dissection of neuronal circuits. In the model organism *Drosophila melanogaster*, several methods for discerning the neurotransmitter systems are available. Here, we expanded the toolbox for the identification of cholinergic neurons by generating a new line FRT-STOP-FRT-VAChT::HA that is a conditional tagged knock-in of the vesicular acetylcholine transporter (*VAChT*) gene in its endogenous locus. Importantly, in comparison to already available tools for the detection of cholinergic neurons, the FRT-STOP-FRT-VAChT::HA allele also allows for identification of the subcellular localization of the cholinergic presynaptic release sites in a cell-specific manner. We used the newly generated FRT-STOP-FRT-VAChT::HA line to characterize the Mi1 and Tm3 neurons in the fly visual system and found that VAChT is present in the axons of both cell types, suggesting that Mi1 and Tm3 neurons provide cholinergic input to the elementary motion detectors, the T4 neurons.

## Abstract (precis)

**Summary:** A new transgenic *Drosophila melanogaster* line for the cell-type-specific identification of cholinergic release sites expands the available methods toolbox for discerning the neurotransmitter systems in the fly nervous system.

## INTRODUCTION

Understanding of the information processing in neuronal circuits requires knowledge about connectivity and properties of the cells involved. The type of neurotransmitter released by a cell defines, to a large extent, the role of a cell and the range of logical operations that are performed within a circuit. Thus, the identification of the cellular neurotransmitter phenotype is of crucial importance for the functional dissection of neuronal circuits.

Various techniques have been described for the identification of neurotransmitter systems in the *Drosophila melanogaster* nervous system. The most common approach is the detection of neurotransmitter molecules (Monastirioti et al., 1995; Yuan et al., 2005; Kolodziejczyk et al., 2008), neurotransmitter-synthesizing enzymes (Takagawa and Salvaterra, 1996; Featherstone et al., 2000; Blanco et al., 2011) or vesicular neurotransmitter transporters (Kitamoto et al., 1998; Daniels et al., 2004; Greer et al., 2005; Romero-Calderón et al., 2008; Fei et al., 2010) with an antibody. In *Drosophila* neurons, the major disadvantage of this strategy is that either cell bodies or larger neuropil areas are examined for the antibody staining. Because of the small diameter of the neuronal processes, reliable localization of the antibody staining within the individual neurites is beyond the resolution threshold of traditional confocal microscopy. Therefore, the markers that localize to the presynaptic regions and are not present ubiquitously in the cytoplasm or at the cytoplasmic membrane of soma cannot be easily detected in individual neurons.

The second approach for the identification of neurotransmitter phenotype is the detection of mRNA transcripts for neurotransmitter-synthesizing enzymes or neurotransmitter vesicular transporters. The *in situ* hybridization technique has been used to study gene expression mainly in fly embryos but also in other tissues, including the nervous system. Nevertheless, the demanding process of probe optimization poses a challenge and therefore this technique is not routinely used to assess neurotransmitter phenotype. With respect to the specificity and dynamic range, the current method of choice for transcript profiling is RNA-seq of a single cell or a homogeneous population of cells (Henry et al., 2012; Thomas et al., 2012). In addition, other techniques such as RT-PCR or gene expression microarrays have been successfully used to study gene expression in *Drosophila* neurons (Nagoshi et al., 2010; Takemura et al., 2011). Regardless of the specific technique, the cell-type-specific transcriptome profiling requires isolation of labeled somata, nuclei or ribosomes in sufficient quantity and purity, which is labor-intensive. Also, contamination of the analyzed sample with mRNA from other cell types may occur during this process.

The third approach relies on genetic labeling of neurons expressing the neurotransmitter-synthesizing enzymes or neurotransmitter vesicular transporters via insertion of a transgene into 5′ UTR or a coding intron of the respective gene (Venken et al., 2011; Diao et al., 2015). When the inserted transgene is a transcription factor of a binary expression system such as Gal4/UAS (Brand and Perrimon, 1993) or LexA/lexAop (Lai and Lee, 2006), the complete expression pattern of a particular gene can be easily identified throughout the whole nervous system. Recently, a set of LexA knock-in lines for the neurotransmitter vesicular transporter genes was generated by ends-out homologous recombination (Simpson, 2016).

Acetylcholine is a major excitatory neurotransmitter in the *Drosophila* nervous system. Synthesis of acetylcholine is catalyzed by the enzyme choline acetyltransferase (ChAT) and its loading into synaptic vesicles is mediated by the vesicular acetylcholine transporter (VAChT). Currently, the available tools for identification of the cholinergic neurons are ChAT antiserum (Takagawa and Salvaterra, 1996), ChAT Trojan-MiMIC driver lines (Venken et al., 2011; Diao et al., 2015) and VAChT-LexA knock-in line (Simpson, 2016).

In the present study, we describe a newly generated FRT-STOP-FRT-VAChT::HA allele for the reporting of the endogenous expression of VAChT that not only identifies neurons with the cholinergic phenotype but also provides information about the subcellular localization of the cholinergic presynaptic release sites.

## MATERIALS AND METHODS

### Fly stocks and genotypes

The flies were raised on a standard cornmeal-agar food at 25°C. The following stocks were used: yw, Act5C-cas9, lig4 (provided by F. Schnorrer, Max Planck Institute of Neurobiology, Germany) (Zhang et al., 2014), UAS-FLP (BDSC 4539 and 8208), UAS-mCD8::GFP (BDSC 5137) (Lee and Luo, 1999), VT25965-Gal4 (T4/T5 line) (provided by B. Dickson, Janelia Research Campus, USA); R20D01-Gal4 (LPi3-4 line) (BDSC 48889) (Jenett et al., 2012); VT7747-AD, VT49371-DBD (Mi1 line) (Ammer et al., 2015), GMRSS00300-split Gal4 (Tm3 line) (provided by A. Nern, Janelia Research Campus, USA), MB008B (Aso et al., 2014), MB112C (Aso et al., 2014), UAS-nsyb::GFP (BDSC 6921) (Zhang et al., 2002) and Act5C-Gal4 (BDSC 4414).

The genotypes of flies used in this study are detailed in Table 1.

**Table 1.**
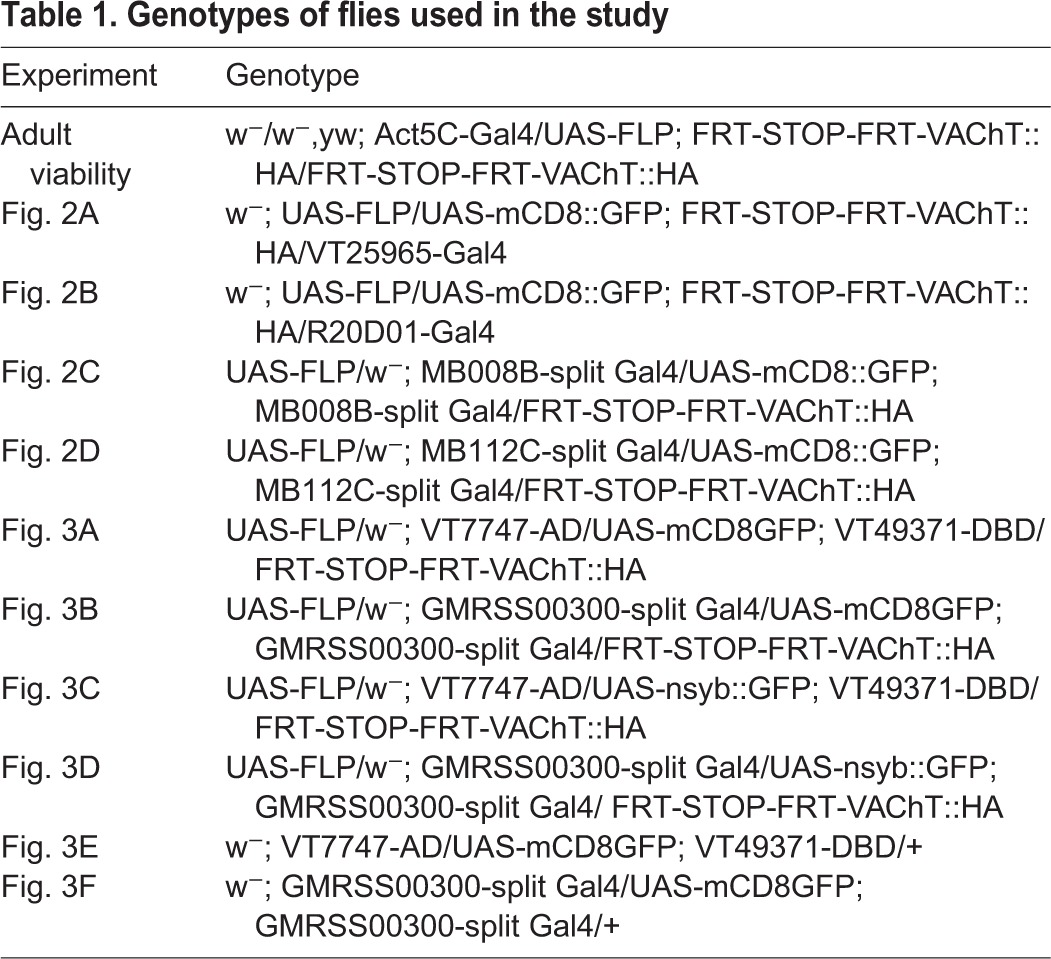
**Genotypes of flies used in the study**

### Generation of the FRT-STOP-FRT-VAChT::HA allele with the CRISPR/cas9 system

The target sites for CRISPR/cas9-induced cleavages in the *VAChT* gene were designed using a web-based software tool (http://crispr.mit.edu/; Hsu et al., 2013). The efficiency of individual guide RNAs (gRNAs) was tested in S2 cells stably expressing cas9 (provided by F. Schnorrer) (Böttcher et al., 2014) as described previously (Zhang et al., 2014). The CRISPR target sites used for genome editing were AGAGGAAGTCCCAAAGAAAC (TGG) and GGGCTATCGATACAATCACG (AGG). The target-specific sequences were cloned into pU6-BbsI-gRNA plasmid (provided by M. Harrison, K. O'Connor-Giles and J. Wildonger; Addgene plasmid 45946) (Gratz et al., 2013) such that the first base of both sequences was replaced by G. The gRNA-expressing plasmids and the donor plasmid for the homology-directed repair were injected into fly embryos of the genotype yw, Act5C-cas9, lig4. The embryo injections were performed by BestGene Inc. (https://www.thebestgene.com/).

The donor fragment for the generation of FRT-STOP-FRT-VAChT::HA allele was assembled by PCR fusion of the following sequences: (1) flippase recognition target (FRT)-flanked cassette containing transcriptional terminator (hsp70Ab polyadenylation signal) and the sequence for a screenable eye marker (3xP3-DsRed-α1tub_3′UTR), synthesized *de novo*; (2) DNA fragment containing the Kozak sequence followed by an open reading frame (ORF) of the *VAChT* gene with the sequence for the HA tag inserted after the first 14 codons from translational start, synthesized *de novo*; and (3) two 1 kb homology arms flanking the CRISPR cleavage sites, amplified from genomic DNA of yw, Act5C-cas9, lig4 flies. The resulting donor fragment consisted of the upstream homology arm fused to the FRT cassette, followed by the Kozak sequence, the ORF with tag sequence and the downstream homology arm. The donor fragment was blunt-end cloned into pJet1.2 vector (Thermo Fisher Scientific). The nucleotide sequence of the HA tag was TAC CCA TAC GAT GTT CCA GAT TAC GCT.

### Immunohistochemistry

Fly brains were dissected in PBS and fixed in 4% PFA with 0.1% Triton X for 25 min. Brains were washed in 0.3% PBT and incubated first with primary (24–72 h) and then secondary (24–48 h) antibodies in 0.3% PBT supplemented with 5% NGS. The brains were mounted in Vectashield mounting medium (Vector Laboratories) and imaged on a Leica TCS SP5 or SP8 laser-scanning confocal microscope. The following antibodies were used: rabbit anti-GFP (Torrey Pines TP401, 1:400), rat anti-HA (Sigma-Aldrich, clone 3F10, 1:100), mouse anti-ChAT (DSHB, deposited by P. Salvaterra, 1:50) (Takagawa and Salvaterra, 1996), goat anti-rabbit Alexa 488 (Thermo Fisher Scientific A-11008, 1:200), goat anti-rat Alexa 647 (Thermo Fisher Scientific A-21247, 1:200) and goat anti-mouse Alexa 647 (Thermo Fisher Scientific A-21235, 1:200).

## RESULTS AND DISCUSSION

Using CRISPR/cas9-based genome editing (Jinek et al., 2012; Gratz et al., 2013), we generated a new allele of the *VAChT* gene that carried an additional HA tag. This new allele was positioned in the original genomic locus of the *VAChT* gene and therefore its expression depended on the endogenous regulatory sequences of *VAChT*. The HA tag was placed after the first 14 amino acids from the N terminus, within the cytoplasmic domain of the VAChT protein. The position of the tag was chosen such that it would not interfere with protein folding or signaling sequences known to participate in the intracellular trafficking of VAChT (Fei et al., 2008).

**Fig. 1.**
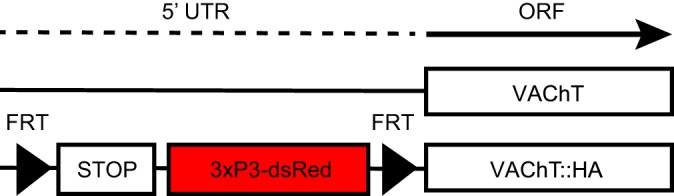
**Original VAChT allele and FRT-STOP-FRT-VAChT::HA allele.** FRT-flanked transcriptional stop signal in the 5′ UTR constrains the expression of VAChT::HA. Removal of the stop cassette requires FLP recombinase, introduced by the Gal4/UAS system. Expression of the VAChT::HA is therefore restricted to cells with active endogenous regulatory sequences of *VAChT* that are, in addition, part of the Gal4 expression pattern. The 3xP3-dsRed sequence encodes a screenable eye marker. ORF, open reading frame.

Our aim was to restrict the expression of VAChT::HA to a population of neurons defined by the expression pattern of a Gal4 line of choice. Therefore, we included a transcriptional stop cassette into the 5′ UTR of the *VAChT* gene that was flanked by two FRT sites (Fig. 1). The expression of the VAChT::HA was, as a result, confined to cells that were expressing flippase (FLP) recombinase introduced by the Gal4/UAS system and contained an active endogenous promoter of *VAChT*.

Disruption of both copies of the *VAChT* gene causes lethality during embryonic or larval development (Kitamoto et al., 2000). We did not observe any adult flies homozygous for the newly generated FRT-STOP-FRT-VAChT::HA allele, confirming that the stop cassette efficiently disrupts transcription of the VAChT::HA. When the stop cassette was removed by expressing the FLP recombinase ubiquitously with Act5C-Gal4 driver line, the flies homozygous for FRT-STOP-FRT-VAChT::HA allele were viable. This suggests that the tagged transporter VAChT::HA can fully substitute the original VAChT transporter at the synapse.

**Fig. 2.**
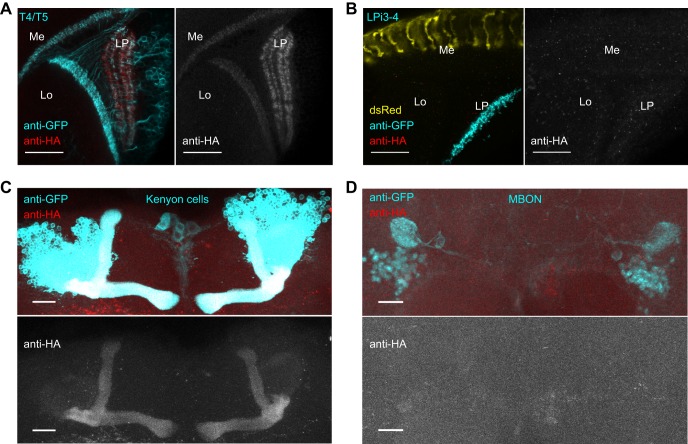
**VAChT::HA is detectable exclusively in the cholinergic neurons.** (A) The expression of VAChT::HA in T4/T5 neurons is localized to the axons in the lobula plate and dendrites in the medulla and lobula. (B) In the LPi3-4 neurons, no expression of VAChT::HA can be detected. The fluorescence of DsRed in the R7/R8 photoreceptor terminals in the medulla confirms the presence of the FRT-STOP-FRT-VAChT::HA allele in the fly genome. (C) Kenyon cells in the α/β lobes of the mushroom body show expression of VAChT::HA. (D) No co-localization of the HA signal and GFP staining in the mushroom body output neurons (MBON) γ1pedc>α/β can be detected. All scale bars: 20 µm. Me, medulla; Lo, lobula; LP, lobula plate.

To test the functionality of the FRT-STOP-FRT-VAChT::HA allele, we chose the T4/T5 neurons. T4/T5 neurons are the elementary motion detectors of the fly, sensitive to motion of bright (T4) and dark (T5) edges (Maisak et al., 2013). These cells have been shown previously to synthesize and release acetylcholine (Mauss et al., 2014; Shinomiya et al., 2014). In accordance with these prior findings, we detected VAChT::HA in the axon terminals of the T4/T5 neurons in the lobula plate (Fig. 2A). A weaker HA signal was present also in the dendrites of T4/T5 neurons in medulla and lobula. This finding is in line with a previous study reporting existence of the dendritic presynaptic release sites in the T4 neurons (Takemura et al., 2013). To show that the expression of VAChT::HA is absent in non-cholinergic neurons, we looked at the expression of VAChT::HA in the LPi3-4 neurons that have been previously identified as glutamatergic (Mauss et al., 2015). As expected, we could not detect any expression of the VAChT::HA in the LPi3-4 neurons (Fig. 2B).

To demonstrate that FRT-STOP-FRT-VAChT::HA allele reliably captures the endogenous expression pattern of the *VAChT* gene in a variety of neuronal populations, we additionally examined the expression of VAChT::HA in the mushroom body neurons. We detected VAChT::HA in the Kenyon cells in α/β lobes of the mushroom body (Fig. 2C) that have recently been shown to release acetylcholine (Barnstedt et al., 2016). On the contrary, we did not observe any HA signal in the GABAergic mushroom body output neurons γ1pedc>α/β (Aso et al., 2014) (Fig. 2D).

Mi1 and Tm3 neurons synapse onto dendrites of T4 neurons (Takemura et al., 2013) and are involved in visual detection of the moving bright edges (Behnia et al., 2014; Ammer et al., 2015). Despite the functional characterization of the responses of Mi1 and Tm3 neurons (Behnia et al., 2014) and the reported effects of the synaptic silencing of Mi1 and Tm3 on the motion vision circuit (Ammer et al., 2015), the exact contribution of the Mi1 and Tm3 neurons to direction-selective responses of the T4 neurons is not clear (Maisak et al., 2013; Haag et al., 2016), nor is it known whether the synaptic input that Mi1 and Tm3 neurons provide to T4 neurons is excitatory or inhibitory. Therefore, we employed the newly generated FRT-STOP-FRT-VAChT::HA allele to investigate the neurotransmitter system used by Mi1 and Tm3 neurons.

**Fig. 3.**
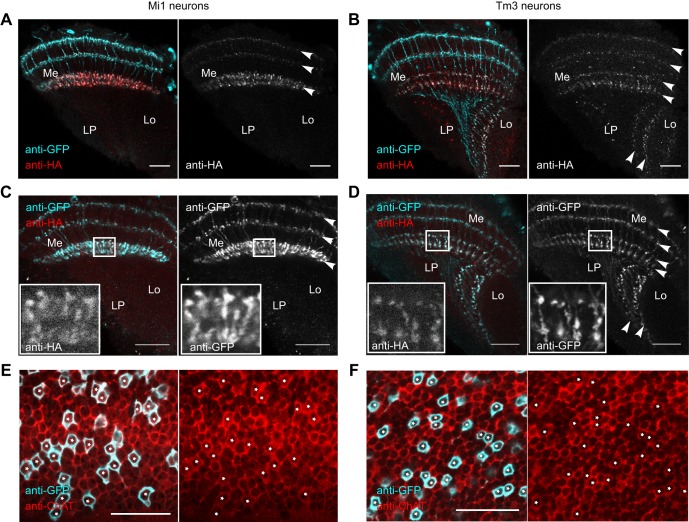
**The Mi1 and Tm3 neurons are cholinergic.** The VAChT::HA can be found in all layers of the medulla and lobula in which Mi1 (A) and Tm3 (B) neurons laterally extend their processes (white arrowheads). The subcellular localization of the presynaptic marker nsyb::GFP in the Mi1 (C) and Tm3 (D) neurons (white arrowheads) corresponds to that of VAChT::HA. The insets in C and D show close-ups of the medulla regions containing presynaptic release sites of Mi1 and Tm3 neurons labeled by VAChT::HA and nsyb::GFP. Anti-ChAT staining co-localizes with the GFP-labeled somatic cytoplasmic membrane of the Mi1 (E) and Tm3 (F) neurons. White asterisks mark the position of the GFP-labeled cell bodies of the Mi1 and Tm3 neurons. All scale bars: 20 µm. Me, medulla; Lo, lobula; LP, lobula plate.

Using the FRT-STOP-FRT-VAChT::HA line, we identified the Mi1 and Tm3 neurons as cholinergic. The expression of VAChT::HA could be detected in all medullar and lobular layers where Mi1 and Tm3 neurons laterally extend their neurites (Fig. 3A,B). The VAChT::HA signal was strongest for both the Mi1 and Tm3 neurons in the medullar layer 9/10, where Mi1 and Tm3 neurons synapse on the dendrites of T4 neurons (Takemura et al., 2013). In order to examine whether the localization of VAChT::HA corresponds to the presynaptic release sites in the Mi1 and Tm3 neurons, we expressed a marker for presynaptic sites, the GFP-tagged neuronal synaptobrevin (nsyb::GFP) (Zhang et al., 2002), in Mi1 and Tm3 neurons. We observed that the subcellular localization of nsyb::GFP in both Mi1 and Tm3 neurons shows the same pattern as VAChT::HA (Fig. 3C,D), confirming that the subcellular distribution of VAChT::HA corresponds to the presynaptic release sites. The expression of nsyb::GFP in Mi1 and Tm3 neurons was stronger than the expression of VAChT::HA and could be detected also in the neuronal fibers. This is likely due to overexpression of the nsyb::GFP transgene with the Gal4/UAS system. To prove the cholinergic phenotype of the Mi1 and Tm3 neurons by another line of evidence, we stained fly brains with ChAT antiserum (Takagawa and Salvaterra, 1996) and looked at the presence of ChAT immunostaining in the cell bodies of Mi1 and Tm3 neurons. We detected the presence of ChAT immunoreactivity in the cell bodies of both Mi1 and Tm3 neurons (Fig. 3E,F), confirming that Mi1 and Tm3 neurons use acetylcholine as their neurotransmitter.

When using the FRT-STOP-FRT-VAChT::HA allele, one important aspect to consider is choosing a Gal4 line with as specific an expression pattern as possible. Even very weak expression of the FLP can lead to genomic excision of the FRT-flanked transcriptional stop cassette and, as a result, to expression of the VAChT::HA. As the expression level of VAChT::HA depends on the endogenous regulatory sequences and not on the amount of Gal4 molecules present, it may occur that the expression of VAChT::HA is stronger than that of Gal4-driven GFP. When using a Gal4 line containing cells with various strength of Gal4 expression, we noticed the presence of VAChT::HA also in the neurons that were barely detectably labeled with GFP. We believe that this is the reason for the unspecific dotted pattern of the anti-HA staining in the optic lobe of the Tm3 line (Fig. 3B).

The decision about which approach to use for the identification of cholinergic neurons should be based on the driver line inspected. For the Gal4 lines with a narrow expression pattern and the split-Gal4 lines, the FRT-STOP-FRT-VAChT::HA allele is the tool of choice. When using the FRT-STOP-FRT-VAChT::HA allele, there is no need for further experiments to determine which neurites contain presynaptic release sites. In contrast, for the Gal4 lines with a broader expression pattern, the examined neurons should instead be tested for co-localization with the expression pattern of the ChAT Trojan-MiMIC driver line (Venken et al., 2011; Diao et al., 2015), the VAChT-LexA knock-in line (Simpson, 2016) or with ChAT antiserum (Takagawa and Salvaterra, 1996).

A previously reported method for synaptic tagging with recombination using bruchpilot protein as a general marker of presynaptic release sites (Chen et al., 2014) served as an inspiration for the generation of the transgenic allele described in this study. The combination of the conditionally tagged bruchpilot protein and the conditionally tagged VAChT might enable enumeration of the total presynaptic release sites and cholinergic release sites simultaneously in a single neuron, assuming that a specific and sparse Gal4 line is provided.

## References

[JEB149369C1] Ammer G., Leonhardt A., Bahl A., Dickson B. J. and Borst A. (2015). Functional specialization of neural input elements to the *Drosophila* ON motion detector. *Curr. Biol.* 25, 2247-2253. 10.1016/j.cub.2015.07.014 26234212

[JEB149369C2] Aso Y., Hattori D., Yu Y., Johnston R. M., Iyer N. A., Ngo T. T., Dionne H., Abbott L. F., Axel R., Tanimoto H. et al. (2014). The neuronal architecture of the mushroom body provides a logic for associative learning. *Elife* 3, e04577 10.7554/eLife.04577 25535793 PMC4273437

[JEB149369C3] Barnstedt O., Owald D., Felsenberg J., Brain R., Moszynski J.-P., Talbot C. B., Perrat P. N. and Waddell S. (2016). Memory-relevant mushroom body output synapses are cholinergic. *Neuron* 89, 1237-1247. 10.1016/j.neuron.2016.02.015 26948892 PMC4819445

[JEB149369C4] Behnia R., Clark D. A., Carter A. G., Clandinin T. R. and Desplan C. (2014). Processing properties of ON and OFF pathways for *Drosophila* motion detection. *Nature* 512, 427-430. 10.1038/nature13427 25043016 PMC4243710

[JEB149369C5] Blanco J., Pandey R., Wasser M. and Udolph G. (2011). Orthodenticle is necessary for survival of a cluster of clonally related dopaminergic neurons in the *Drosophila* larval and adult brain. *Neural Dev.* 6, 34 10.1186/1749-8104-6-34 21999236 PMC3206411

[JEB149369C6] Böttcher R., Hollmann M., Merk K., Nitschko V., Obermaier C., Philippou-Massier J., Wieland I., Gaul U. and Förstemann K. (2014). Efficient chromosomal gene modification with CRISPR/cas9 and PCR-based homologous recombination donors in cultured *Drosophila* cells. *Nucleic Acids Res.* 42, e89 10.1093/nar/gku289 24748663 PMC4066747

[JEB149369C7] Brand A. H. and Perrimon N. (1993). Targeted gene expression as a means of altering cell fates and generating dominant phenotypes. *Development* 118, 401-415. 8223268 10.1242/dev.118.2.401

[JEB149369C8] Chen Y., Akin O., Nern A., Tsui C. Y. K., Pecot M. Y. and Zipursky S. L. (2014). Cell-type-specific labeling of synapses *in vivo* through synaptic tagging with recombination. *Neuron* 81, 280-293. 10.1016/j.neuron.2013.12.021 24462095 PMC4025979

[JEB149369C9] Daniels R. W., Collins C. A., Gelfand M. V., Dant J., Brooks E. S., Krantz D. E. and DiAntonio A. (2004). Increased expression of the *Drosophila* vesicular glutamate transporter leads to excess glutamate release and a compensatory decrease in quantal content. *J. Neurosci.* 24, 10466-10474. 10.1523/JNEUROSCI.3001-04.2004 15548661 PMC6730318

[JEB149369C10] Diao F., Ironfield H., Luan H., Diao F., Shropshire W. C., Ewer J., Marr E., Potter C. J., Landgraf M. and White B. H. (2015). Plug-and-play genetic access to *Drosophila* cell types using exchangeable exon cassettes. *Cell Rep.* 10, 1410-1421. 10.1016/j.celrep.2015.01.059 25732830 PMC4373654

[JEB149369C11] Featherstone D. E., Rushton E. M., Hilderbrand-Chae M., Phillips A. M., Jackson F. R. and Broadie K. (2000). Presynaptic glutamic acid decarboxylase is required for induction of the postsynaptic receptor field at a glutamatergic synapse. *Neuron* 27, 71-84. 10.1016/S0896-6273(00)00010-6 10939332

[JEB149369C12] Fei H., Grygoruk A., Brooks E. S., Chen A. and Krantz D. E. (2008). Trafficking of vesicular neurotransmitter transporters. *Traffic* 9, 1425-1436. 10.1111/j.1600-0854.2008.00771.x 18507811 PMC2897747

[JEB149369C13] Fei H., Chow D. M., Chen A., Romero-Calderón R., Ong W. S., Ackerson L. C., Maidment N. T., Simpson J. H., Frye M. A. and Krantz D. E. (2010). Mutation of the *Drosophila* vesicular GABA transporter disrupts visual figure detection. *J. Exp. Biol.* 213, 1717-1730. 10.1242/jeb.036053 20435823 PMC2861964

[JEB149369C14] Gratz S. J., Cummings A. M., Nguyen J. N., Hamm D. C., Donohue L. K., Harrison M. M., Wildonger J. and O'Connor-Giles K. M. (2013). Genome engineering of *Drosophila* with the CRISPR RNA-guided Cas9 nuclease. *Genetics* 194, 1029-1035. 10.1534/genetics.113.152710 23709638 PMC3730909

[JEB149369C15] Greer C. L., Grygoruk A., Patton D. E., Ley B., Romero-Calderon R., Chang H.-Y., Houshyar R., Bainton R. J., Diantonio A. and Krantz D. E. (2005). A splice variant of the *Drosophila* vesicular monoamine transporter contains a conserved trafficking domain and functions in the storage of dopamine, serotonin, and octopamine. *J. Neurobiol.* 64, 239-258. 10.1002/neu.20146 15849736

[JEB149369C16] Haag J., Arenz A., Serbe E., Gabbiani F. and Borst A. (2016). Complementary mechanisms create direction selectivity in the fly. *Elife* 5, e17421 10.7554/eLife.17421 27502554 PMC4978522

[JEB149369C17] Henry G. L., Davis F. P., Picard S. and Eddy S. R. (2012). Cell type-specific genomics of *Drosophila* neurons. *Nucleic Acids Res.* 40, 9691-9704. 10.1093/nar/gks671 22855560 PMC3479168

[JEB149369C18] Hsu P. D., Scott D. A., Weinstein J. A., Ran F. A., Konermann S., Agarwala V., Li Y., Fine E. J., Wu X., Shalem O. et al. (2013). DNA targeting specificity of RNA-guided Cas9 nucleases. *Nat. Biotechnol.* 31, 827-832. 10.1038/nbt.2647 23873081 PMC3969858

[JEB149369C19] Jenett A., Rubin G. M., Ngo T.-T. B., Shepherd D., Murphy C., Dionne H., Pfeiffer B. D., Cavallaro A., Hall D., Jeter J. et al. (2012). A GAL4-driver line resource for *Drosophila* neurobiology. *Cell Rep.* 2, 991-1001. 10.1016/j.celrep.2012.09.011 23063364 PMC3515021

[JEB149369C20] Jinek M., Chylinski K., Fonfara I., Hauer M., Doudna J. A. and Charpentier E. (2012). A programmable dual-RNA-guided DNA endonuclease in adaptive bacterial immunity. *Science* 337, 816-821. 10.1126/science.1225829 22745249 PMC6286148

[JEB149369C21] Kitamoto T., Wang W. and Salvaterra P. M. (1998). Structure and organization of the *Drosophila* cholinergic locus. *J. Biol. Chem.* 273, 2706-2713. 10.1074/jbc.273.5.2706 9446576

[JEB149369C22] Kitamoto T., Xie X., Wu C.-F. and Salvaterra P. M. (2000). Isolation and characterization of mutants for the vesicular acetylcholine transporter gene in *Drosophila melanogaster*. *J. Neurobiol.* 42, 161-171. 10.1002/(SICI)1097-4695(20000205)42:2<161::AID-NEU1>3.0.CO;2-P 10640324

[JEB149369C23] Kolodziejczyk A., Sun X., Meinertzhagen I. A. and Nässel D. R. (2008). Glutamate, GABA and acetylcholine signaling components in the lamina of the *Drosophila* visual system. *PLoS ONE* 3, e2110 10.1371/journal.pone.0002110 18464935 PMC2373871

[JEB149369C24] Lai S. L. and Lee T. (2006). Genetic mosaic with dual binary transcriptional systems in *Drosophila*. *Nat. Neurosci.* 9, 703-709. 10.1038/nn1681 16582903

[JEB149369C25] Lee T. and Luo L. (1999). Mosaic analysis with a repressible cell marker for studies of gene function in neuronal morphogenesis. *Neuron* 22, 451-461. 10.1016/S0896-6273(00)80701-1 10197526

[JEB149369C26] Maisak M. S., Haag J., Ammer G., Serbe E., Meier M., Leonhardt A., Schilling T., Bahl A., Rubin G. M., Nern A. et al. (2013). A directional tuning map of *Drosophila* elementary motion detectors. *Nature* 500, 212-216. 10.1038/nature12320 23925246

[JEB149369C27] Mauss A. S., Meier M., Serbe E. and Borst A. (2014). Optogenetic and pharmacologic dissection of feedforward inhibition in *Drosophila* motion vision. *J. Neurosci.* 34, 2254-2263. 10.1523/JNEUROSCI.3938-13.2014 24501364 PMC6608528

[JEB149369C28] Mauss A. S., Pankova K., Arenz A., Nern A., Rubin G. M. and Borst A. (2015). Neural circuit to integrate opposing motions in the visual field. *Cell* 162, 351-362. 10.1016/j.cell.2015.06.035 26186189

[JEB149369C29] Monastirioti M., Gorczyca M., Rapus J., Eckert M., White K. and Budnik V. (1995). Octopamine immunoreactivity in the fruit fly *Drosophila melanogaster*. *J. Comp. Neurol.* 356, 275-287. 10.1002/cne.903560210 7629319 PMC4664080

[JEB149369C30] Nagoshi E., Sugino K., Kula E., Okazaki E., Tachibana T., Nelson S. and Rosbash M. (2010). Dissecting differential gene expression within the circadian neuronal circuit of *Drosophila*. *Nat. Neurosci.* 13, 60-68. 10.1038/nn.2451 19966839 PMC3878269

[JEB149369C31] Romero-Calderón R., Uhlenbrock G., Borycz J., Simon A. F., Grygoruk A., Yee S. K., Shyer A., Ackerson L. C., Maidment N. T., Meinertzhagen I. A. et al. (2008). A glial variant of the vesicular monoamine transporter is required to store histamine in the *Drosophila* visual system. *PLoS Genet.* 4, e1000245 10.1371/journal.pgen.1000245 18989452 PMC2570955

[JEB149369C32] Simpson J. H. (2016). Rationally subdividing the fly nervous system with versatile expression reagents. *J. Neurogenet.* 30, 185-194. 10.1080/01677063.2016.1248761 27846759

[JEB149369C33] Shinomiya K., Karuppudurai T., Lin T.-Y., Lu Z., Lee C.-H. and Meinertzhagen I. A. (2014). Candidate neural substrates for off-edge motion detection in *Drosophila*. *Curr. Biol.* 24, 1062-1070. 10.1016/j.cub.2014.03.051 24768048 PMC4031294

[JEB149369C34] Takagawa K. and Salvaterra P (1996). Analysis of choline acetyltransferase protein in temperature sensitive mutant flies using newly generated monoclonal antibody. *Neurosci. Res.* 24, 237-243. 10.1016/0168-0102(95)00999-X 8815444

[JEB149369C35] Takemura S. Y., Karuppudurai T., Ting C. Y., Lu Z., Lee C. H. and Meinertzhagen I. A. (2011). Cholinergic circuits integrate neighboring visual signals in a *Drosophila* motion detection pathway. *Curr. Biol.* 21, 2077-2084. 10.1016/j.cub.2011.10.053 22137471 PMC3265035

[JEB149369C36] Takemura S. Y., Bharioke A., Lu Z., Nern A., Vitaladevuni S., Rivlin P. K., Katz W. T., Olbris D. J., Plaza S. M., Winston P. et al. (2013). A visual motion detection circuit suggested by *Drosophila* connectomics. *Nature* 500, 175-181. 10.1038/nature12450 23925240 PMC3799980

[JEB149369C37] Thomas A., Lee P.-J., Dalton J. E., Nomie K. J., Stoica L., Costa-Mattioli M., Chang P., Nuzhdin S., Arbeitman M. N. and Dierick H. A. (2012). A versatile method for cell-specific profiling of translated mRNAs in Drosophila. *PLoS ONE* 7, e40276 10.1371/journal.pone.0040276 22792260 PMC3391276

[JEB149369C38] Venken K. J. T., Schulze K. L., Haelterman N. A., Pan H., He Y., Evans-Holm M., Carlson J. W., Levis R. W., Spradling A. C., Hoskins R. A. et al. (2011). MiMIC: a highly versatile transposon insertion resource for engineering *Drosophila melanogaster* genes. *Nat. Methods* 8, 737-743. 10.1038/nmeth.1662 21985007 PMC3191940

[JEB149369C39] Yuan Q., Lin F., Zheng X. and Sehgal A. (2005). Serotonin modulates circadian entrainment in Drosophila. *Neuron* 47, 115-127. 10.1016/j.neuron.2005.05.027 15996552

[JEB149369C40] Zhang X., Koolhaas W. H. and Schnorrer F. (2014). A versatile two-step CRISPR- and RMCE-based strategy for efficient genome engineering in *Drosophila*. *G3* 4, 2409-2418. 10.1534/g3.114.013979 25324299 PMC4267936

[JEB149369C41] Zhang Y. Q., Rodesch C. K. and Broadie K. (2002). Living synaptic vesicle marker: synaptotagmin-GFP. *Genesis* 34, 142-145. 10.1002/gene.10144 12324970

